# Impact of dobutamine stress on diastolic energetic efficiency of healthy left ventricle: an *in vivo* kinetic energy analysis

**DOI:** 10.3389/fcvm.2023.1103751

**Published:** 2023-03-21

**Authors:** Alessandra Riva, Jonatan Eriksson, Federica Viola, Francesco Sturla, Emiliano Votta, Tino Ebbers, Carl-Johan Gustav Carlhäll, Petter Dyverfeldt

**Affiliations:** ^1^ Department of Electronics, Information and Bioengineering, Politecnico di Milano, Milan, Italy; ^2^ 3D and Computer Simulation Laboratory, IRCCS, Policlinico San Donato, San Donato Milanese, Italy; ^3^ Center for Medical Image Science and Visualization, Linköping University, Linköping, Sweden; ^4^ Department of Medical Radiation Physics and Department of Health, Medicine and Caring Sciences, Linköping University, Linköping, Sweden; ^5^ Unit of Cardiovascular Sciences, Department of Health, Medicine and Caring Sciences, Linköping University, Linköping, Sweden; ^6^ Department of Clinical Physiology in Linköping, Department of Health, Medicine and Caring Sciences, Linköping University, Linköping, Sweden

**Keywords:** 4D flow MRI, dobutamine stress, kinetic energy, left ventricle, turbulent kinetic energy, flow physiology, stress cardiovascular magnetic resonance, hemodynamics

## Abstract

The total kinetic energy (KE) of blood can be decomposed into mean KE (MKE) and turbulent KE (TKE), which are associated with the phase-averaged fluid velocity field and the instantaneous velocity fluctuations, respectively. The aim of this study was to explore the effects of pharmacologically induced stress on MKE and TKE in the left ventricle (LV) in a cohort of healthy volunteers. 4D Flow MRI data were acquired in eleven subjects at rest and after dobutamine infusion, at a heart rate that was ∼60% higher than the one in rest conditions. MKE and TKE were computed as volume integrals over the whole LV and as data mapped to functional LV flow components, i.e., direct flow, retained inflow, delayed ejection flow and residual volume. Diastolic MKE and TKE increased under stress, in particular at peak early filling and peak atrial contraction. Augmented LV inotropy and cardiac frequency also caused an increase in direct flow and retained inflow MKE and TKE. However, the TKE/KE ratio remained comparable between rest and stress conditions, suggesting that LV intracavitary fluid dynamics can adapt to stress conditions without altering the TKE to KE balance of the normal left ventricle at rest.

## Introduction

Turbulent flow exhibits spatiotemporal velocity fluctuations, as well as chaotic and unsteady vortical structures at different scales. Non-turbulent vortices are relatively long lasting, orderly, and continuous. In turbulent flow, relatively small vortices can quickly appear and disappear and have varying lifetime, vortex size, and density (1, 2).

Turbulence can be assessed through Reynolds decomposition, in which velocity is separated into phase-averaged (i.e., mean) velocity and time-varying fluctuating velocity, where the latter is characterized by fast apparently random velocity fluctuations. Similarly, the total fluid kinetic energy (KE) can be decomposed into mean KE (MKE) and turbulent KE (TKE) (3). MKE is a function of the mean fluid velocity field and intensifies in regions with high velocities. TKE is characterized by the root mean square of the fast velocity fluctuations. Because TKE is dissipated through energy transfer to small eddies, it is frequently used as an index of flow inefficiency associated with turbulent features (4–7).

Intracardiac blood flow is predominantly laminar under normal physiological conditions (8). However, several cardiovascular diseases and intracavitary dysfunctions may facilitate blood flow transition into turbulence (9, 10), with altered blood flow being associated with hemolysis, platelet activation and thrombus formation (11–13). Turbulent blood flow can be investigated *in vitro,* using laser doppler or particle image velocimetry on mock circulation loops (14, 15), *in silico*, including the effect of turbulence in computational fluid dynamic simulations (16, 17), and *in vivo*, by three-dimensional (3D) time-resolved phase contrast cardiac magnetic resonance (MR) with three-directional velocity encoding (4D Flow MRI). Through dedicated sequences, 4D Flow MRI allows the quantification of turbulent intensity of small-scale velocity fluctuations in cardiovascular flows and of mean velocity, thus enabling the quantification of TKE and MKE from one MRI acquisition (4, 5, 9, 18, 19).

4D Flow MRI offers the unique advantage to estimate *in vivo* blood flow turbulence in healthy subjects and patients, avoiding simplifications, required by *in vitro* mock loops (20), as well as assumptions and input parameters required by *in silico* approaches (21). Also, the total 4D Flow MRI scan time is limited (∼10 min) and data post-processing, despite non-trivial, is significantly less demanding if compared to the time-expense required by computational simulations. 4D Flow MRI is still far from reaching the spatial and temporal granularity of numerical simulations, however its application is increasingly and rapidly expanding to investigate a wide spectrum of cardiovascular disorders, offering a reasonable trade-off between reliability and uncertainty of the analysis (22).

Several studies have employed 4D Flow MRI to investigate *in vivo* left ventricle (LV) intracavitary flow and elucidated the hemodynamics derangements associated with different patterns of LV dysfunctions. Intra-cardiac energetics has been quantified largely at rest, revealing altered ventricular MKE in several cardiovascular diseases (23, 24). A few studies also focused on LV hemodynamic changes during physical or pharmacological cardiac stress tests, which increase heart rate (HR), LV cardiac output, the contractile strength of heart muscle (i.e., inotropy) and the proportion of ventricular work converted to MKE (25–28). In a previous study, dobutamine-induced cardiac stress caused an increase in the fraction of LV volume associated to the direct flow (DF) component, i.e., blood entering and leaving the LV in the same cardiac cycle, as well as an increase in MKE for each of the four LV flow components, this effect being the greatest for DF (25). This previous study speculated that this increase in MKE reflects an improved efficiency of LV blood flow transit in the setting of dobutamine-generated increased inotropy and cardiac frequency.

Herein we test that speculation by exploring if the dobutamine-induced increase in diastolic LV blood flow MKE is accompanied by a corresponding increase in TKE, which would reveal a transition from laminar to turbulent blood flow, detrimental to LV blood flow efficiency (29). This new analysis is aimed to contribute to the understanding of the mechanisms that allow the LV to adapt to stress conditions. To this end, 4D Flow MRI data were acquired in healthy subjects at rest and under dobutamine-induced cardiac stress and were used to compare MKE and TKE between rest and stress conditions for the whole LV chamber and LV flow components.

## Methods

### Study population

Fourteen subjects were enrolled in the study. No history of and no medication for cardiovascular disease as well as a normal physical examination were required to be included in the study. Accordingly, exclusion criteria include abnormal LV size, wall thickness or wall motion from cardiac MRI at rest (30), more than moderate arterial hypertension (blood pressure at rest > 150/90 mmHg), acute coronary disease, severe aortic stenosis and hypertrophic obstructive cardiomyopathy. The study was approved by the Regional Ethical Review Board in Linköping and complies with the Declaration of Helsinki. All subjects provided written informed consent.

### Study plan

MRI scans were performed at rest and after dobutamine infusion. Dobutamine dose was set as 5–10 µg/kg/min and adjusted every 2 min, until reaching a HR ∼60% higher than the one at rest. This target HR was chosen in order to achieve HR in the range of those from previous studies on stress testing with MRI (31–34). HR was monitored continuously throughout the study and blood pressures were measured at rest and after dobutamine. The dobutamine infusion was maintained until both MR and 4D Flow MRI data under stress conditions were acquired.

### Data acquisition

MRI scans were acquired on a 3 T Philips Ingenia scanner (Philips Healthcare, Best, the Netherlands). The imaging protocol comprised short- and long-axis *cine* balanced steady-state free precession (bSSFP) images (three-chamber and four-chamber at rest, three-chamber only under dobutamine) reconstructed to 30 timeframes throughout the cardiac cycle. The bSSFP images were acquired during end-expiratory breath-holds with the following scan parameters: echo time (TE) = 1.4–1.5 ms, repetition time (TR) = 2.7–3.0 ms, flip angle = 45°, pixel spacing = (0.9–1.0) × (0.9–1.0) mm^2^, slice thickness = 8 mm.

The 4D Flow MRI data were acquired during free breathing, using a navigator-gated gradient-echo pulse-sequence and retrospective gating. Scan parameters were: velocity encoding (VENC) = 120–150 cm/s, flip angle = 5°, TE = 3 ms, TR = 5.1–5.2 ms, parallel imaging (SENSE) speed up factors = 3 (AP direction), ×1.6 (RL direction), k-space segmentation factor = 2, elliptical k-space acquisition, acquired and reconstructed spatial resolution = (2.3–2.7) × (2.3–2.7) × 2.8 mm^3^, effective temporal resolution = 40.8–41.6 ms, which was reconstructed to 40 timeframes. Asymmetric four-point flow encoding was used to obtain velocity as well as intravoxel standard deviation data. Scan time was approximately 7–8 min including navigator efficiency. 4D Flow MRI velocity data were corrected for concomitant gradient fields on the scanner, as well as background phase-offsets due to eddy currents and phase wraps during post-processing (35, 36).

The magnitude images of the individual flow-encoding segments were reconstructed to compute TKE per unit volume as (37):

(1) $$TKE=\frac{1}{2}\rho \overset{3}{\underset{i=1}{\sum }}\sigma_{i}^{2}\;\;\left[\frac{J}{m^{3}}\right]$$

where ρ is the blood density (1.060 kg/m^3^), and $\;\sigma_{i}$ is the velocity fluctuation intensity in three orthogonal directions. For the asymmetric four-point flow encoding, $\;\sigma_{i}$ was obtained as (4, 5):

(2) $$\sigma_{i}=\frac{1}{k_{v}}\sqrt{2\frac{\left|S\right|}{\left|S_{i}\right|}}\;\left[\frac{m}{s}\right]$$

where |S| and $\left|S_{i}\right|$ are the magnitude of MR signal with and without motion sensitivity encoding, respectively. $k_{v}$ represents the motion sensitivity and is defined as:

(3) $$k_{v}=\frac{\pi }{VENC}\;\left[\frac{s}{m}\right]$$

TKE data were filtered by a 3 × 3 × 3 median filter to reduce noise.

### Data analysis

The data were analyzed by computing the diastolic and peak MKE and TKE in the LV volume, as well as the MKE and TKE in the different flow components, which reflects the MKE and TKE the blood experiences along their trajectory through the LV. Furthermore, the TKE/KE ratio was computed for both approaches.

To accomplish this, the LV was segmented throughout the entire cardiac cycle in the cine short-axis images at rest and after dobutamine, using Segment (v1.9, Medviso, Lund, Sweden) (38). Papillary muscles and trabeculae were considered as part of the LV cavity volume (i.e., blood pool). End-systolic (ES) and end-diastolic (ED) volume, stroke volume, ejection fraction, and cardiac output were computed. The entire processing pipeline was implemented through *ad hoc* scripts coded in Matlab (MathWorks, Natick, USA). LV segmentations were resampled to match the spatial and temporal resolution of the 4D Flow MRI data spatial and temporal resolution. At each time point, global LV TKE was computed by integrating the TKE in all the voxels in the LV segmentation. To account for the effect of LV size, TKE values were normalized to the current LV (TKE_V_). Likewise, MKE was computed for the whole chamber as:

(4) $$MKE=\overset{N}{\underset{i=1}{\sum }}\frac{1}{2}\rho V_{i}v_{i}^{2}$$

where $V_{i}$ the $i^{th}$ voxel volume, *v* the velocity magnitude and *N* the total number of voxels within the LV. At each time-point, global MKE was then indexed to the current volume of LV (MKE_V_).

LV flow components were computed using a consolidated method described previously (39) and whose accuracy has already been tested (25, 40). Briefly, pathlines were emitted from each voxel inside the LV at ED and traced forward and backward in time until ES. ED and ES were defined as the timeframes corresponding to the largest and the smallest ventricular volumes, respectively. Pathlines were separated into four flow components, according to their route through the LV (Supplementary Figure S1): *direct flow* (DF), blood that enters the LV during diastole and leaves during systole in the analyzed heartbeat; *retained inflow* (RI), blood that enters the LV but does not leave in the analyzed heartbeat; *delayed ejection flow* (DE), blood that resides in the LV during diastole and leaves during systole in the analyzed heartbeat; *residual volume* (RV), blood that resides in the LV for at least two cardiac cycles. Pathlines that did not meet any of these criteria were classified as *non-physiological flow*. Subjects with more than 15% non-physiological flow in at least one dataset were excluded from the study. Additionally, subjects with LV inflow vs. LV outflow discrepancy > 15% in at least one dataset were excluded as well (see Supplementary Material). In addition to the global LV TKE, the TKE of the subvolume of blood associated with each flow component was computed as follows (Supplementary Figure S2):

(1) The trajectory of each pathline was downsampled so as to obtain the Lagrangian positions corresponding to the reconstructed 4D Flow MRI time frames (i.e., 40) for the acquired cardiac cycle;
(2) A value of TKE was assigned to each point of the pathline at each time frame using trilinear space-interpolation:
  (5) $$TKE_{\;pathlinepoint}^{t}=\frac{\sum_{\;j=1}^{8}w_{j}\cdot V_{j}\cdot TKE_{j}^{t}}{\sum_{\;j=1}^{8}w_{j}}$$
  where $TKE_{j}^{t}$ is the TKE in the $j^{th}$ voxel at timeframe *t* and $w_{j}$ is a weight function defined as:
  (6) $$w_{j}=e^{-\frac{{\left|\;p_{j}-d\right|}^{2}}{{\left(d/2\right)}^{2}}}\;with\;j=1,\ldots ,8$$
  where *d* is the voxel diagonal, $p_{j}$ is the distance between the pathline point and the $j^{th}$ voxel center. The summation was always extended to the 8 voxels closest to the pathline position at time *t*. This choice guaranteed that $TKE_{\;pathlinepoint}^{t}$ accounted for the contribution of at least one voxel on each side of the pathline point, while reducing computational effort.
(3) Finally, for each flow component, TKE was integrated over the cardiac cycle for each pathline belonging to that flow component and subsequently normalized by the volume of each flow component (FC), yielding $TKE_{V}^{FC}$, where FC = DF, RI, DE and RV.

The MKE of flow components normalized by the component volume ($MKE_{V}^{FC}$) was computed as described in (40).

Peak E-wave (i.e., early diastolic filling) and peak A-wave (i.e., late diastolic filling) were identified based on the time course of MKE_V_ as the two highest values throughout diastole, respectively. Volumetric and FC-specific TKE_V_ and MKE_V_ values were extracted at these time-points. Temporal curves for all computed variables were also integrated over time throughout diastole.

As a way of exploring the TKE relative to the total KE of the flow, the TKE/KE ratio was computed as:

(7) $$\frac{TKE}{KE}=\frac{TKE}{TKE+MKE}$$

The TKE/KE ratio represents the portion of total KE dissipated due to turbulence and thus it is an index of energetic efficiency. The ratio was evaluated for all computed variables, considering the instantaneous values at peak E-wave and peak A-wave, as well as the time integrals over the diastolic phase.

### Statistical analysis

All results are reported as mean ± SD, unless otherwise stated. Normality of distribution of continuous data was assessed through the Shapiro-Wilk test. Data were compared using t-test, if normally distributed, and using Wilcoxon signed-rank test, otherwise. Statistical analysis was performed with GraphPad Prism 8 (GraphPad Software Inc., La Jolla, CA, USA); a *p*-value < 0.05 was considered significant.

## Results

Three subjects were excluded due to inflow-outflow discrepancies >15% or non-physiological flow percentage >15% under dobutamine. Demographic and morphological characteristics for the study population are detailed in Table 1. Pharmacologically induced stress resulted in a significant increase in HR, EF and CO, and significant decrease in LV volumes. Moreover, dobutamine infusion significantly shortened the diastolic phase.

**Table 1:** Demographical and clinical data for the study population.

	Rest (*n* = 11)	Dobutamine (*n* = 11)	*p*-value
Age (years)	28 [23; 42]	–	
Sex (F:M)	7:4	–	
Height (cm)	174 ± 9	–	
Weight (kg)	69 ± 9	–	
BSA (m^2^)	1.82 ± 0.15	–	
HR (bpm)	64 ± 10	105 ± 19	<0.0001
BP systolic (mmHg)	119 ± 11	133 ± 17	0.06
BP diastolic (mmHg)	67 ± 10	78 ± 27	0.30
EDV_i_ (ml/m^2^)	84 ± 13	73 ± 16	0.0008
ESV_i_ (ml/m^2^)	38 ± 9	23 ± 8	<0.0001
SV_i_ (ml/m^2^)	47 ± 7	50 ± 9	0.278
EF (%)	56 ± 6	69 ± 5	0.0002
CI (l/min/m^2^)	3.0 ± 0.5	5.1 ± 0.7	<0.0001
Diastolic length (s)	0.59 ± 0.11	0.36 ± 0.07	<0.0001

Data are expressed as mean ± SD. BP, blood pressure; BSA, body surface area; CI, cardiac index; EDV, end-diastolic volume; ESV, end-systolic volume; HR, heart rate; SV, stroke volume.

### MKE_V_ and TKE_V_ diastolic time integrals

**Figure 1:**
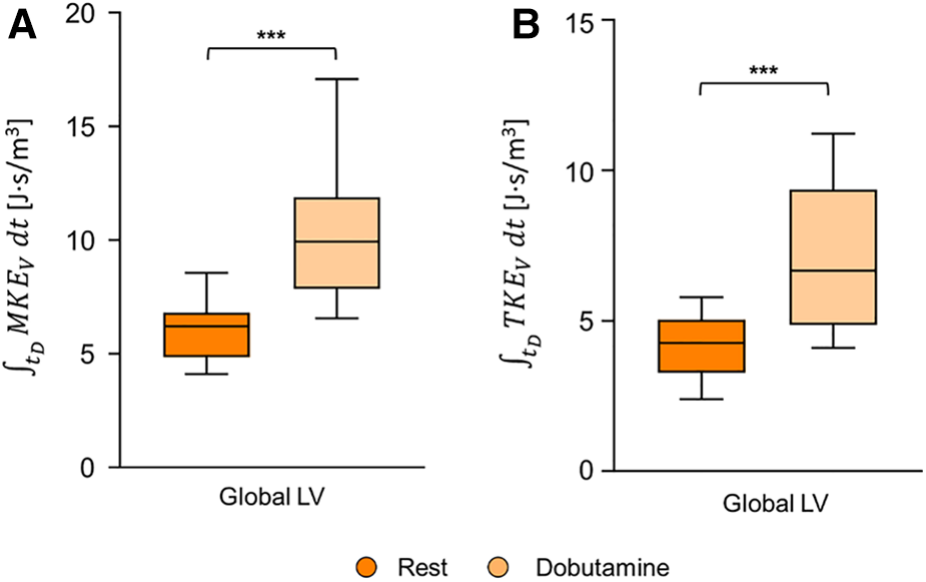
Box and whiskers plot for integral diastolic (**A** MKE_V_ and **B**) TKE_V_ for the whole LV. Each box ranges between 25th and 75th percentile with a line pointing out the median value; whiskers indicate the 10th and the 90th percentile, respectively. *, *p* ≤ 0.05; **, *p* ≤ 0.01; ***, *p* ≤ 0.001; ****, *p* ≤ 0.0001.

Upon dobutamine infusion, the diastolic time integral of MKE_V_ significantly increased for the global LV (Figure 1A). The diastolic time integral of TKE_V_ significantly increased for the whole LV (Figure 1B).

**Figure 2:**
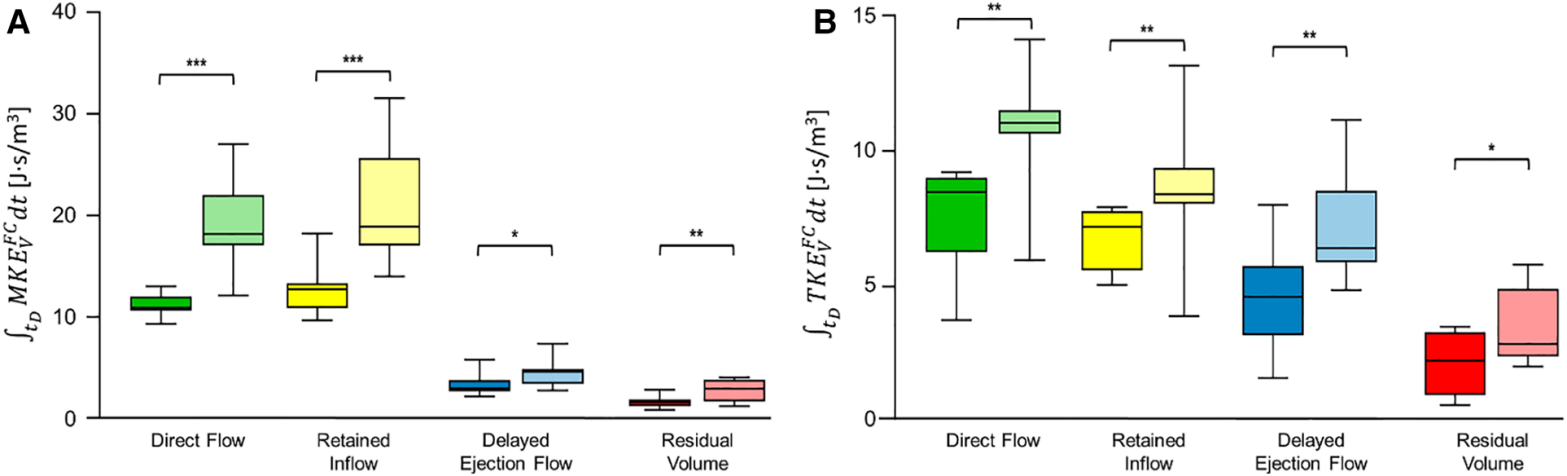
Box and whiskers plot of (**A**) $MKE_{V}^{FC}$ and (**B**) $TKE_{V}^{FC}$ during diastole for each flow component. Box plot in dark color (**left**) represents values at rest and box plot in bright color (**right**) represents values after dobutamine infusion. Each box ranges between 25^th^ and 75^th^ percentile with a line pointing out the median value; whiskers indicate the 10^th^ and the 90^th^ percentile, respectively. *, *p* ≤ 0.05; **, *p* ≤ 0.01; ***, *p* ≤ 0.001. FC, flow component; MKE, mean kinetic energy; TKE, turbulent kinetic energy.

The diastolic time integral of $MKE_{V}^{FC}$ and of $TKE_{V}^{FC}$ significantly increased under stress conditions for all FCs (Figure 2, Supplementary Table S2).

### MKE_V_ and TKE_V_ at peak E-wave and peak A-wave

**Figure 3:**
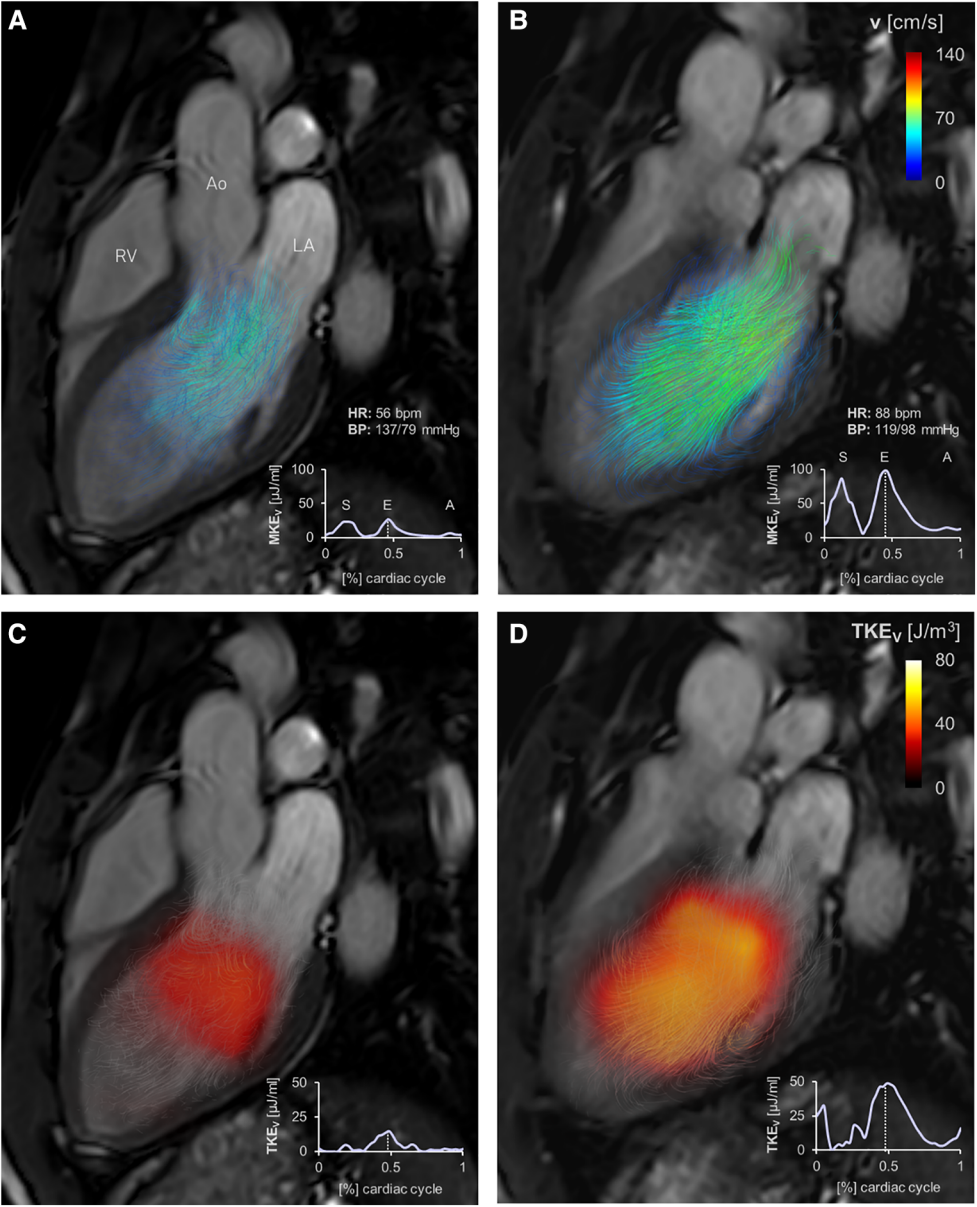
Velocity color-coded streamlines (**A,B**) and TKE_V_ maps (**C,D**) at rest (**left**) and under stress (**right**) represented at peak E-wave (dashed white line) for one of the analyzed subjects. A, peak A-wave; Ao, aorta; BP, blood pressure; E, peak E-wave; HR, heart rate; LA, left atrium; MKE, mean kinetic energy; RV, right ventricle; S, peak systole; TKE, turbulent kinetic energy.

In all subjects, both at rest and upon dobutamine infusion, two peaks in global LV MKE_V_ were observed in diastole: the first peak occurred during the E-wave; the second peak was smaller and occurred during the A-wave (Figures 3A,B). At rest, the two peaks were clearly separated; under dobutamine-induced stress conditions, the A-wave peak was in continuity with the end of the E-wave.

**Figure 4:**
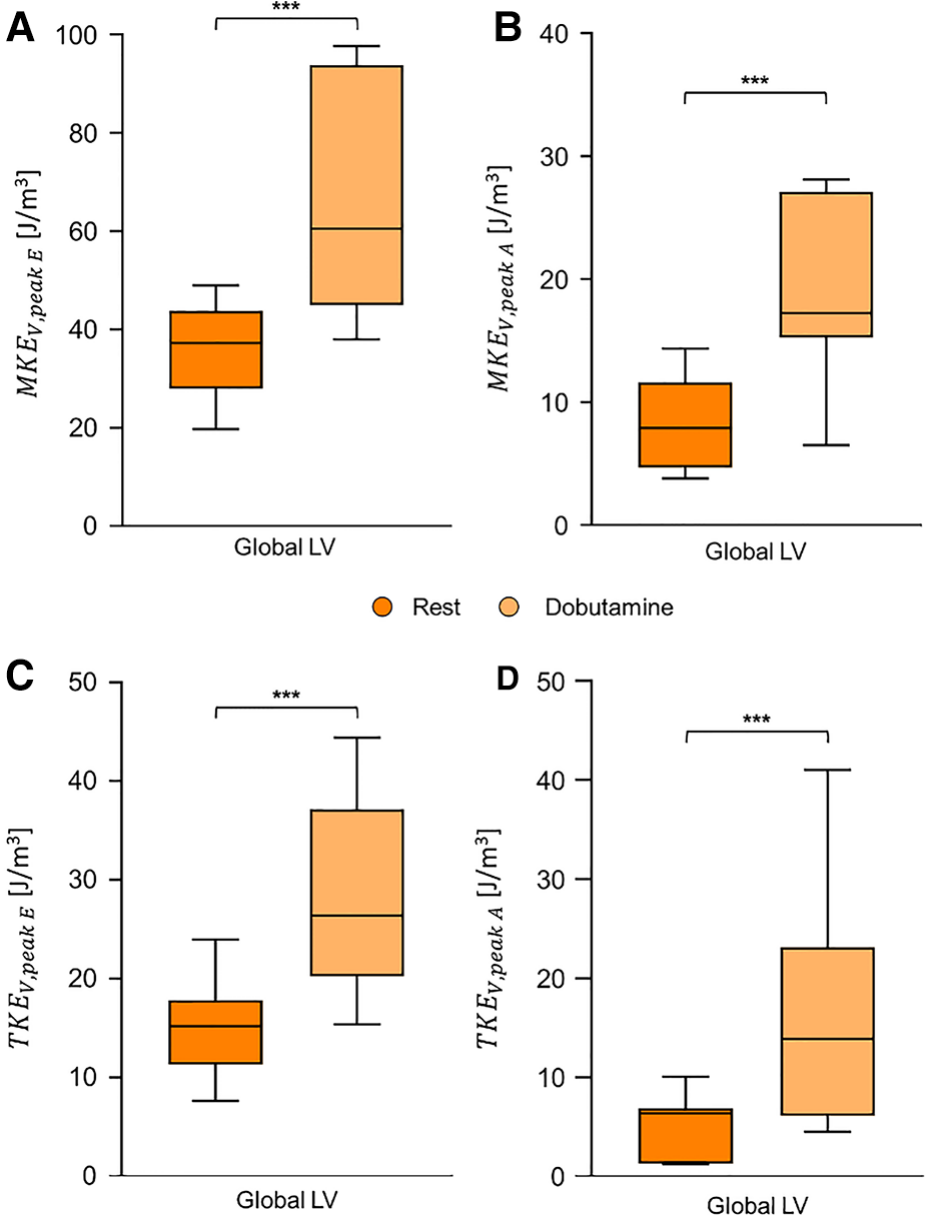
Box and whiskers plot for global LV MKE_V_ at peak E-wave (**A**) and at peak A-wave (**B**) and for global LV TKE_V_ at peak E-wave (**C**) and at peak A-wave (**D**). Each box ranges between 25^th^ and 75^th^ percentile with a line pointing out the median value; whiskers indicate the 10^th^ and the 90^th^ percentile, respectively. *, *p* ≤ 0.05; **, *p* ≤ 0.01; ***, *p* ≤ 0.001; ****, *p* ≤ 0.0001.

Peak E-wave and peak A-wave MKE_V_ significantly increased under stress for global LV (Figures 4A,B).

After dobutamine infusion, peak E-wave and peak A-wave TKE_V_ significantly increased for the entire LV (Figures 4C,D).

### Flow components and associated MKE and TKE

**Figure 5:**
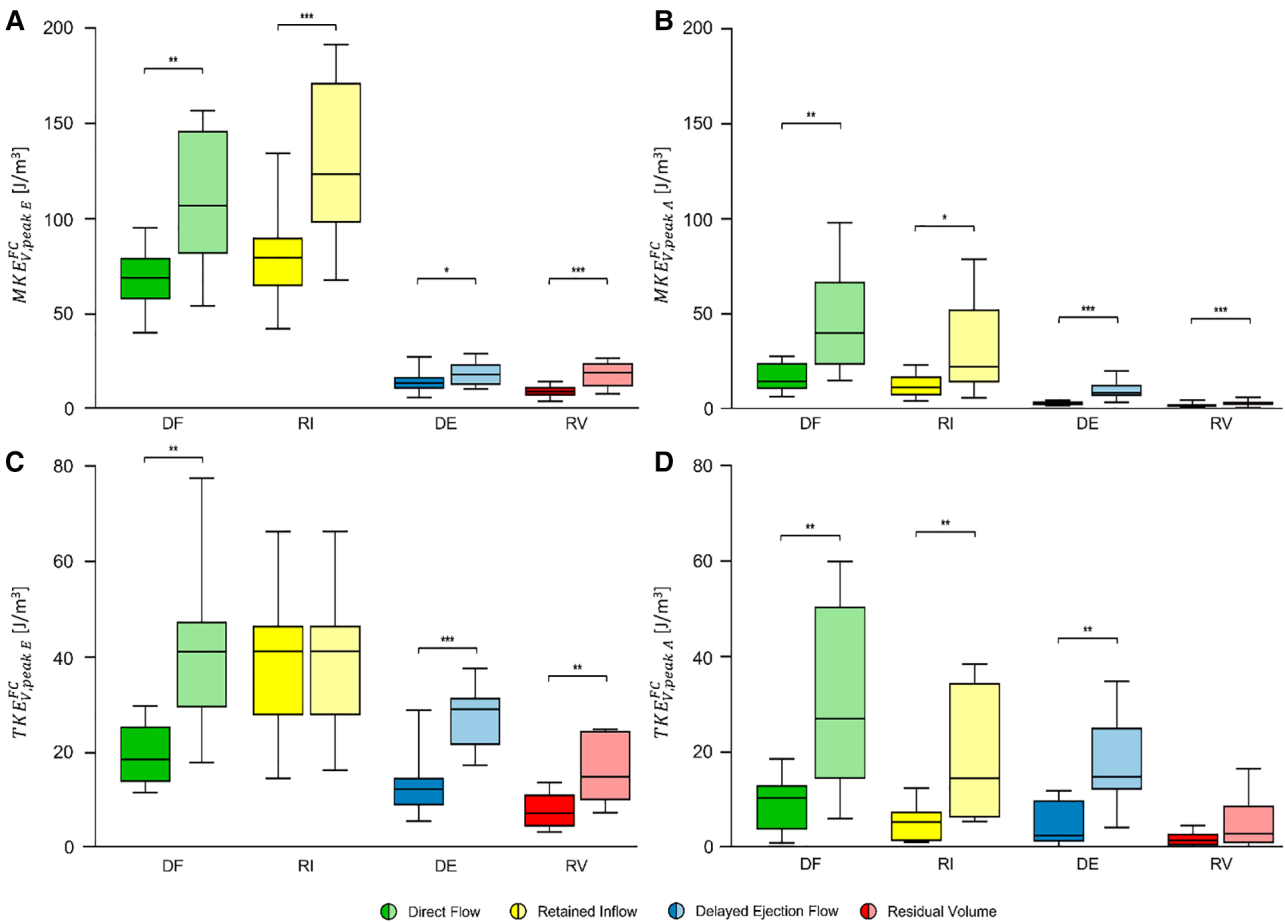
$MKE_{V}^{FC}$ (**top panel**) and $TKE_{V}^{FC}$ (**bottom panel**) [J/m^3^] for each flow component at peak E-wave (**A,C**) and at peak A-wave (**B,D**). Box plot in dark color (**left**) represents values at rest and box plot in bright color (**right**) represents values after dobutamine infusion. Each box ranges between 25^th^ and 75^th^ percentile with a line pointing out the median value; whiskers indicate the 10^th^ and the 90^th^ percentile, respectively. *, *p* ≤ 0.05; **, *p* ≤ 0.01; ***, *p* ≤ 0.001. FC, flow component; DF, direct flow; RI, retained inflow; DE, delayed ejection flow; RV, residual volume; MKE, mean kinetic energy; TKE, turbulent kinetic energy.

Dobutamine infusion induced changes on flow components and on associated kinetic energies, namely:

• altered LV flow subdivision, resulting in significantly increased DF and significantly decreased DE and RV (Supplementary Figure S3);
• significant increase in $MKE_{V}^{FC}$ for every FC at peak E-wave (Figure 5A) and at peak A-wave (Figure 5B);
• significant increase in $TKE_{V}^{DF}$, $TKE_{V}^{DE}$, and $TKE_{V}^{RV}$ but not in $TKE_{V}^{RI}$ (*p* = 0.341) at peak E-wave (Figure 5C, Supplementary Table S1);
• significant increase in $TKE_{V}^{DF}$, $TKE_{V}^{RI}$, and $TKE_{V}^{DE}$, but not in $TKE_{V}^{RV}$ (*p* = 0.083) at peak A-wave (Figure 5D, Supplementary Table S1).

### TKE/KE ratio

**Figure 6:**
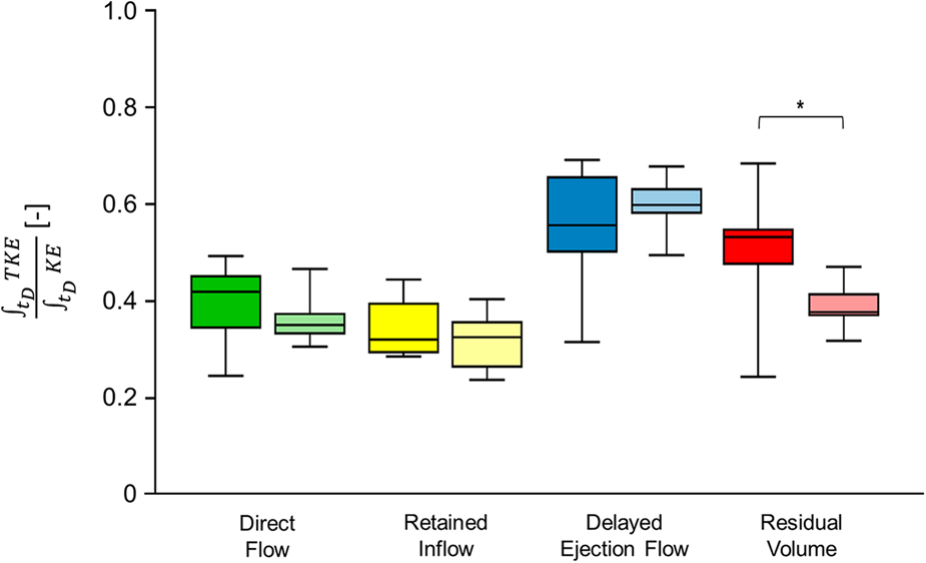
Box and whiskers plot for diastolic time integrals TKE/KE for flow components. Box plot in dark color (**left**) represents values at rest and box plot in bright color (**right**) represents values after dobutamine infusion. Each box ranges between 25^th^ and 75^th^ percentile with a line pointing out the median value; whiskers indicate the 10^th^ and the 90^th^ percentile, respectively. *, *p* ≤ 0.05. FC, flow component; KE, kinetic energy; TKE, turbulent kinetic energy.

Dobutamine infusion did not induce significant changes in the ratio between TKE and KE time-integrals computed for the entire LV (0.4 ± 0.09 at rest, 0.41 ± 0.06 under stress, *p* = 0.62). No significant changes were induced in the ratio between TKE and KE time-integrals associated to flow components, with the exception of RV, which showed a significant decrease in the ratio under stress (Figure 6).

The TKE/KE ratio, evaluated at peak E-wave (0.33 ± 0.08 at rest, 0.34 ± 0.06 under stress) and at peak A-wave (0.40 ± 0.21 at rest, 0.44 ± 0.14 under stress), remained comparable between the two HR conditions for the whole LV (*p* = 0.15 and *p* = 0.37, respectively).

When analyzed based on flow components, the TKE/KE ratio at peak E-wave significantly increased for DF and for DE, and significantly decreased for RI, while no significant differences were found for RV. At peak A-wave, no significant differences were found between rest and stress conditions (Table 2).

**Table 2:** TKE/KE ratio for each flow component at peak E-wave and at peak A-wave.

	Rest (*n* = 11)	Dobutamine (*n* = 11)	*p*-value
**Peak E-wave** TKE//KE **[-]**			
Direct Flow	0.22 ± 0.06	0.28 ± 0.07	0.03
Retained Inflow	0.32 ± 0.10	0.23 ± 0.05	0.001
Delayed Ejection Flow	0.47 ± 0.12	0.59 ± 0.09	0.002
Residual Volume	0.42 ± 0.12	0.47 ± 0.11	0.24
**Peak A-wave** TKE//KE **[-]**			
Direct Flow	0.37 ± 0.21	0.38 ± 0.15	0.81
Retained Inflow	0.31 ± 0.16	0.37 ± 0.15	0.38
Delayed Ejection Flow	0.46 ± 0.26	0.62 ± 0.13	0.05
Residual Volume	0.44 ± 0.26	0.48 ± 0.25	0.78

Data are expressed as mean ± SD. KE, kinetic energy; TKE, turbulent kinetic energy.

## Discussion

The effect of dobutamine-induced stress on MKE and TKE was investigated non-invasively using 4D Flow MRI in a cohort of healthy volunteers by measuring MKE, TKE and the TKE/KE ratio throughout diastole for the whole LV and for flow components. Resembling moderate exercise conditions, dobutamine infusion led to significantly increased HR, cardiac output and ejection fraction and to significantly decreased end-diastolic volume, end-systolic volume and diastolic length. Moreover, it generally caused a significant increase in MKE and TKE throughout diastole generally for global and compartmental LV flow. However, the TKE/KE ratio was similar for the two HR conditions in the analyzed cohort.

### Rest condition energetics

Under physiologic rest conditions, human LV function is often considered to be closer to maximal efficiency when compared to pathologic rest conditions. LV efficiency depends on multiple factors, including myofibers metabolic efficiency and mechanics as well as intracavitary fluid dynamics. For the latter, efficiency is typically analyzed focusing on LV systolic pump function only and no established criteria are available to elucidate the potential role of each factor.

For instance, when considering LV wall myofiber mechanics, the Frank-Starling law states that there is an optimal end-diastolic length between sarcomeres at which the tension in the muscle fiber is maximized (41). Also, the tension generated by myofibers, which is linked to intracavitary pressure, increases as their rate of shortening, which is linked to heart rate, decreases (42). Consequently, the mechanical power per unit volume produced by myofibers is maximal for intermediate values of stress and strain rate, and hence of intracavitary pressure and heart rate.

When considering intracavitary LV blood flow, clear criteria are not yet established and different flow features may be considered depending on whether efficiency is analyzed over the whole cycle, the systolic phase or the diastolic phase. In the present study, we analyzed intracavitary flow components during the cardiac cycle and the analysis of diastolic blood flow MKE and TKE, and the TKE/KE ratio, in an attempt to gain insight into diastolic LV flow efficiency in rest and stress-induced conditions.

Previous studies suggest that physiological rest conditions are characterized by distinct proportions of intracardiac flow components (39). DF follows the most efficient path (i.e., shortest and fastest) for optimal flow ejection into the systemic circulation. MKE was mainly stored by the DF and RI flow components, in accordance with previous studies (24, 43). DF pathlines follow an efficient pathway to the LVOT, characterized by shortest distance, more favorable angle and conserved linear momentum as compared to the other flow components (44). DF and RI decelerate at the end of diastole and then acquire additional MKE prior to being ejected during the subsequent systole (45). As previously reported (24, 43), MKE associated to RI could be transferred to DE and RV, converted into potential energy (either stored within the elastic recoil of the myocardium or elevating ventricular pressure) or dissipated. As for MKE, TKE was mainly found in DF and RI components. At rest, TKE accounts for a non-negligible part of the total fluid KE (∼30%–40%), as highlighted by the TKE/KE ratio. Of note, this is the first study to report TKE data mapped to flow components, as well as to analyze LV flow efficiency though the combined quantification of MKE and TKE.

### Rest vs. stress conditions

In high-demand situations, such as physical exercise, the working point defined by the Frank-Starling law is shifted, as CO increases, maintaining oxygen supply to muscles. Priority is diverted to LV pumping output and the LV flow pattern might change accordingly (46, 47). In the present study, at higher HR, diastasis between early and late diastolic filling was barely visible; this led to higher MKE conservation throughout diastole. In the studied cohort, MKE significantly increased after dobutamine infusion. MKE also increased for all LV flow components, with the most notable increase for DF and RI, as in (25, 47). While results on MKE confirm previously published data, results on TKE provide new insights. Significantly increased TKE values were found throughout diastole for the whole LV. As observed for MKE, TKE increased for all LV flow components. The combined analysis of both KE components, through the TKE/KE ratio, revealed that the increase in MKE was paired with a proportional increase in TKE and thus TKE/KE was similar for the two HR conditions for most LV flow components and throughout the diastolic phase. Interestingly, dobutamine administration did not alter significantly the TKE/KE ratio at peak A-wave, suggesting that, despite increasing myocardial contractility, it does not affect significantly atrial contraction at late diastole, at least not enough to impact blood energetics during late diastolic filling.

### Preserved TKE/KE ratio of the LV under dobutamine-induced stress

The comprehensive analysis of both KE components has never been addressed before: the combined analysis of MKE and TKE could provide a more exhaustive understanding of hemodynamic phenomena in the LV. This study showed that, under dobutamine-induced stress conditions, MKE increases consistently with the increase in ejection fraction and HR. This increase in MKE was complemented by an increase in TKE, which indicates a greater dissipation of energy. However, the TKE/KE ratio was similar at rest and stress conditions, suggesting that, as far as the analyzed variables are concerned, the efficiency of LV diastolic intracavitary fluid dynamics in the two conditions is similar. Notably, these results are for healthy LVs.

### Clinical perspectives

Some cardiac pathologies may remain asymptomatic, with no notable impact on LV inflow and ejection, under rest conditions. Yet, their presence may be revealed by functional derangements under even mild physical activity such as walking a few stairs. For example, the discrimination between pathological LV hypertrophy, due to early myopathies, and physiological hypertrophy, due to physical training, can be difficult at resting conditions. However, cardiac stress can unmask a LV dysfunction in a heart with pathological hypertrophy, unlike in a heart with physiological hypertrophy. Likewise, a mitral valve that is obstructed to a certain degree, due to rheumatic disease or post intervention for example, may show normal inflow at rest but impaired inflow at cardiac stress.

Along this line, here we speculate that the comparison of LV blood flow features such as the LV TKE/KE ratio in stress conditions vs. rest conditions may reveal the presence of cardiac pathologies, related to LV flow efficiency, that remain subclinical in rest conditions.

## Limitations

The cohort of analyzed subjects is relatively small and participants spanned a wide range of age.

Stress conditions were induced by dobutamine and not obtained through real physical exercise. This approach provided better quality of imaging under stress-induced conditions, if compared to physical exercise, and could be easily extended to real patients, including those who could not perform physical exercise. However, dobutamine administration makes the test semi-invasive due to the need for an intravenous catheter, can make ECG signals difficult to interpret, and though mimicking the effects of physical exercise may not be fully equivalent to it.

Also, pressure data are missing for one subject at rest and for two subjects under dobutamine. VENC ranged from 120 to 150 cm/s, which could lead to some variability in noise. However, VENC was set to 140 cm/s for 9 of 11 subjects and all these relatively high VENC values minimize the risk of TKE underestimation due to noise floor effect in MR magnitude images (6). MRI scans were performed in supine conditions and with still body, hence the results do not replicate the ones in posture conditions (48).

## Conclusion

This 4D Flow MRI study employed a combined analysis of MKE and TKE in intracavitary blood flow, specifically focusing on their relationship between rest and dobutamine-induced stress conditions. MKE and TKE increased in healthy LVs at stress. However, the TKE/KE ratio was similar at rest and stress in the analyzed cohort of normal individuals, suggesting that the energetic efficiency was maintained at stress. If further extended to clinically relevant scenarios, the TKE/KE ratio may provide additional insight into LV pathophysiology.

## Data Availability

The raw data supporting the conclusions of this article will be made available by the authors, without undue reservation.
