# Supplementary material for: Impact of dobutamine stress on diastolic energetic efficiency of healthy left ventricle: an in vivo kinetic energy analysis
Source: Front Cardiovasc Med. 2023 Mar 21;10:1103751. doi: 10.3389/fcvm.2023.1103751 (PMC10071008; doi:10.3389/fcvm.2023.1103751)
Supplement: Supplementary file 1 [file Datasheet1.pdf]

Online supplement to

Impact of dobutamine stress on diastolic energetic efficiency of healthy left ventricle: an *in vivo* kinetic energy analysis

Alessandra Riva[1,2]*, Jonatan Eriksson[3,4], Federica Viola[5], Francesco Sturla[2,1], Emiliano Votta[1,2], Tino Ebbers[5,3], Carl-Johan Carlhäll[5,3,6], Petter Dyvertfeldt[5,3]

[1] Department of Electronics, Information and Bioengineering, Politecnico di Milano, Milan, Italy

[2] 3D and Computer Simulation Laboratory, IRCCS, Policlinico San Donato, San Donato Milanese, Italy

[3] Center for Medical Image Science and Visualization, Linköping University, Sweden

[4] Department of Medical Radiation Physics and Department of Health, Medicine and Caring Sciences, Linköping University, Linköping, Sweden

[5] Unit of Cardiovascular Sciences, Department of Health, Medicine and Caring Sciences, Linköping University, Linköping, Sweden

[6] Department of Clinical Physiology in Linköping, Department of Health, Medicine and Caring Sciences, Linköping University, Linköping, Sweden

Supplementary methods

The discrepancy between LV inflow and outflow, when computing pathlines, was computed as:

$$\frac{Inflow - Outflow}{mean(Inflow, Outflow)} \cdot 100$$

The diastolic length (**Table 1**) was computed as the time difference between ED and ES:

$$diastolic - length\,[s] = t_{ED} - t_{ES}$$

Supplementary Tables

Table S1. TKE_V^{FC} for each flow component, computed during diastole for peak E-wave and A-wave.

	**Rest** (n = 11)	**Dobutamine** (n = 11)	***p*-value**
Peak E-wave - TKE_V^{FC} [J/m^3]			
Direct Flow	19.4 ± 6.1	42.1 ± 17.5	0.002
Retained Inflow	38.7 ± 15.5	39.5 ± 14.5	0.341
Delayed Ejection Flow	12.4 [8.9; 14.8]	27.2 ± 6.8	0.001
Residual Volume	7.5 ± 3.8	15.0 [10.0; 24.9]	0.003
Peak A-wave - TKE_V^{FC} [J/m^3]			
Direct Flow	9.9 ± 5.9	31.4 ± 19.7	0.003
Retained Inflow	5.7 ± 3.7	14.7 [6.5; 34.5]	0.003
Delayed Ejection Flow	4.8 ± 4.4	18.2 ± 10.3	0.001
Residual Volume	2.0 ± 1.6	3.1 [1.1; 17.5]	0.083

TKE_V, turbulent kinetic energy normalized per flow component (FC) volume.

Table S2. Time integral of TKE_V^{FC} for each flow component during diastole.

$\int_{t_D} TKE_V^{FC} dt$ [Pa·s]	**Rest** (n = 11)	**Dobutamine** (n = 11)	***p*-value**
Direct Flow	8.50 [6.29; 9.01]	10.7 ± 2.34	0.002
Retained Inflow	6.71 ± 1.14	9.55 ± 2.59	0.004
Delayed Ejection Flow	4.64 ± 2.03	7.08 ± 2.05	0.002
Residual Volume	2.20 ± 1.06	3.54 ± 1.40	0.027

TKE_V, turbulent kinetic energy normalized per flow component (FC) volume.

Supplementary Figures

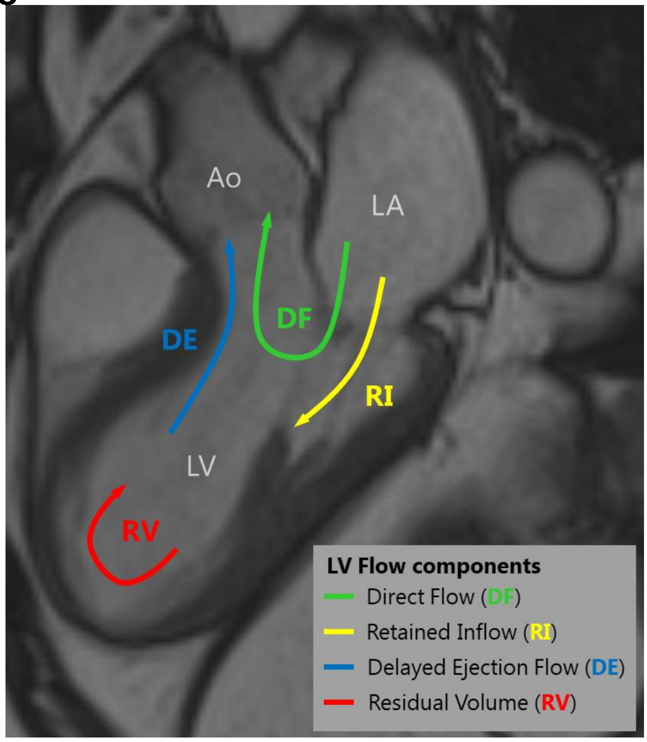

Figure S1. Schematic of the four flow components of the left ventricular blood flow. Direct Flow (DF, green) is the blood flow that enters the left ventricle during diastole and leaves during systole, for the analyzed heartbeat. Retained Inflow (RI, yellow) is the blood that enters the left ventricle during diastole but does not leave during systole in the considered heartbeat. Delayed Ejection Flow (DE, light blue) is the blood that already resides in the left ventricle during diastole and leaves during systole for the analyzed heartbeat. Residual Volume (RV, red) is the blood that resides in the left ventricle for at least two cardiac cycles. Ao, aorta; LA, left atrium; LV, left ventricle.

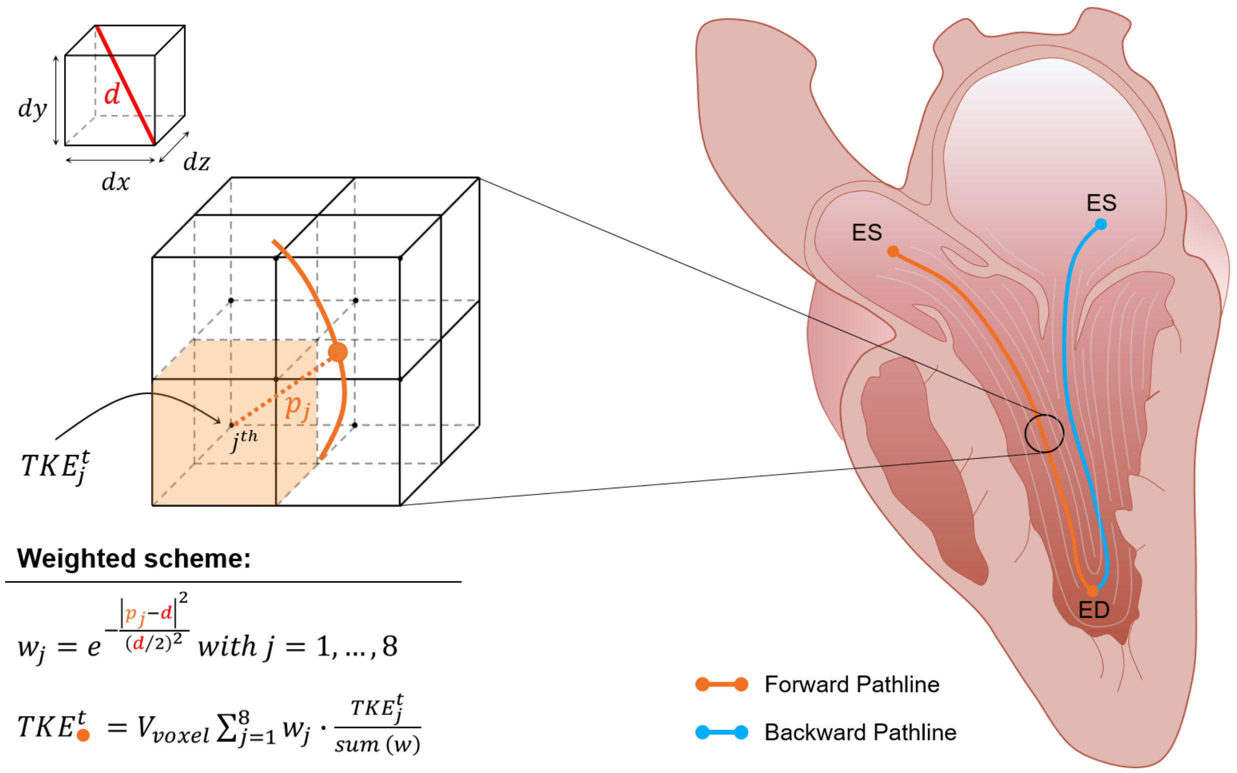

Figure S2. Schematic representation of the exploited weighted scheme for pathline TKE computation. ED, end diastole; ES, end systole; TKE, turbulent kinetic energy.

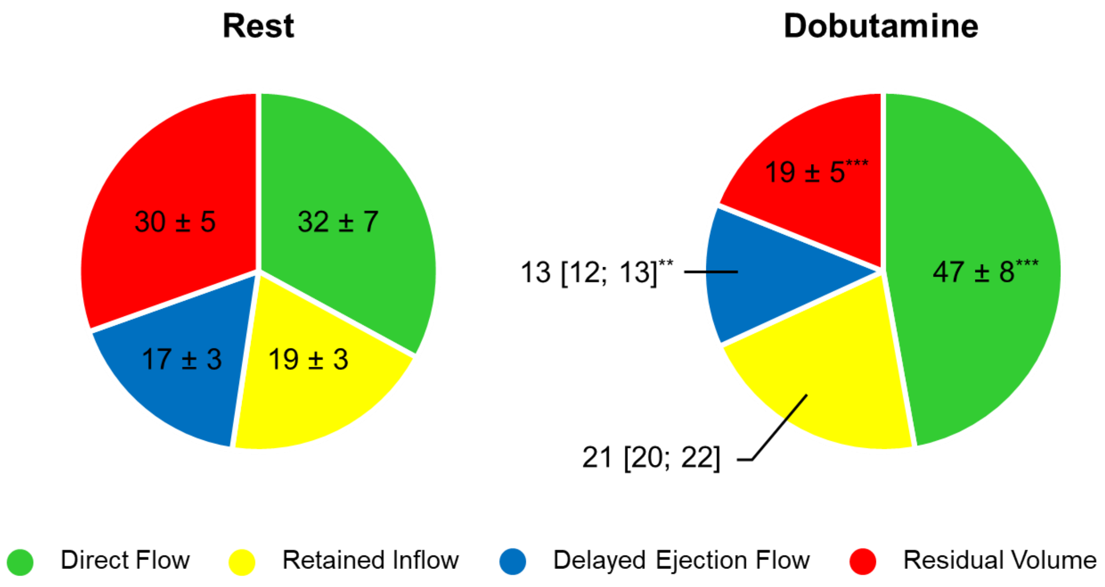

Figure S3. LV flow components as percentage of LV end-diastolic volume expressed as mean ± SD, if normally distributed, as median [IQR] otherwise. *, $p \leq 0.05$; **, $p \leq 0.01$; ***, $p \leq 0.001$.
